# Supplementary material for: Biochemical Characterizations of the Putative Endolysin Ecd09610 Catalytic Domain from Clostridioides difficile
Source: Antibiotics (Basel). 2022 Aug 20;11(8):1131. doi: 10.3390/antibiotics11081131 (PMC9405191; doi:10.3390/antibiotics11081131)
Supplement: Supplementary file 1 [file antibiotics-11-01131-s001.zip › antibiotics-1838430-supplementary.pdf]

Supplementary data

Biochemical Characterizations of the Putative Endolysin Ecd09610 Catalytic Domain from *Clostridioides difficile*

Hiroshi Sekiya [1], Hina Yamaji [1], Ayumi Yoshida [1], Risa Matsunami [1], Shigehiro Kamitori [2] and Eiji Tamai [1,2*]

Figure S1. Multiple sequence alignment of the catalytic domains of Ecd09610.

Figure S2. Species specificity of Ecd09610CD1, Ecd09610CD3, and Ecd09610CD53 lytic activities.

Figure S3. Binding assay for Ecd09610CD1, Ecd09610CD3, and Ecd09610CD53 with various bacteria.

Figure S4. Genes around ecd09610 (CD09610) in the *C. difficile* 630 genome.

Figure S5. Binding ability of the purified N-terminal uncharacterized region of Ecd09610 to *C. difficile* 630 cells.

Figure S6. Effect of NaCl on the binding ability of purified proteins to *C. difficile* 630.

Figure S7. Effect of the two catalytic domains.

Table S1. Primers used in this study.

a

```
                      Accession no.
Ecd09610     371   CAJ67802.1          KVFKNKLSNTGNIFVKYSNAYKVNAALMAAISIHETGNGSSSLCK---------------------------------NK
CWH          371   YP_009206142.1      KLLKGKLSNTGNIFVKYSNAYKVNPALMAAISMHESARGTSNIAN---------------------------------TK
AcpCD         69   5WQW                LSNKGVLTGQGQAFVNAAKAFNIDPIYLVAQCLHETGNGTSKLAKGVTITEIADESKPIYNGNGQLVGYHMIKLSKPVTV
AtlE         104   SIN26952.1          LKGKGILENRGKVFLEAQEKYEVNVIYLVSHALVETGNGKSELAKGIKDGK--------------------------KRY
AtlA-gl       91   6FXP                LVDRPTLLKHTDDFLKAAKDKHVNEVYLISHALLETGAVKSELANGVEIDG--------------------------KKY
StFlgJ         1   5DN4                MDSKDFLARLSLPARLASEQSGVPHHLILAQAALESGWGQRQILRENG-----------------------------EPS
LytB         530   AAK75086.1          NINNSLLENKGATFKEAEEHYHINALYLLAHSALESNWGRSKIAK---------------------------------DK

Ecd09610     419   CAJ67802.1          NNFFGMKG------------------------------MSFGSVDEGIKRGISNLSRNYIHTGRKTLESIRD-KYAPL--
CWH          419   YP_009206142.1      NNFFGMKKNGDY--------------------------MSFSSVDEGIKRGISNLSRNYIHIGRKTLESIRN-KYS----
AcpCD        150   5WQW                YNLFGIGAKDNSSVFPNRALILGTTYAYN---------RGWTSIENAIKGAAEFVSLNYVHSSRYSQNTLYKMRYNQNVS
AtlE         159   SIN26952.1          YNFFGIGAFDSSA------VRSGKSYAEK---------EQWTSPDKAIIGGAKFIRNEYFEN---NQLNLYQMRWNPENP
AtlA-gl      146   6FXP                YNFYGVGALDKDP------IKTGAEYAKK---------HGWDTPEKAISGGADFIHKHFLSS--TDQNTLYSMRWNPKNP
StFlgJ        53   5DN4                YNVFGVKATASWKGPVT--EITTTEYENGEAKKVKAKFRVYSSYLEALSDYVALLTRN----PRYAAVTTAATAEQGAVA
LytB         578   AAK75086.1          NNFFGITAYDTTP------------YLSA---------KTFDDVDKGILGATKWIKENYIDRGRTF--------LGNKAS

                      Accession no.
Ecd09610     466   CAJ67802.1          ---YDSPLNKDWVPGV----GKFYKQITGNAYSSNS
CWH_g        468   YP_009206142.1      -----SSSDKEWVKCV----GAFYKQITGSTYNSNS
AcpCD        221   5WQW                NIWHQYATTPWYASSIADIMRS-YQDLYLEN-----
AtlE         221   SIN26952.1          A-QHQYASDIRWADKIAKLMDKSYKQFGIKK-----
AtlA-gl      209   6FXP                G-EHQYATDIKWAESNATIIADFYKNMKTEG-----
StFlgJ       127   5DN4                LQNAGYATDPNYARKLTSMIQQLK-AMSEKVSK---
LytB         629   AAK75086.1          GMNVEYASDPYWGEKIASVMMKINEKLGGKD-----
```

b

```
                      Accession no.
Ecd09610     529   CAJ67802.1          KVSKVIQEAKNQL-GKPYAWGGN-------------GPKSFDCSGLMVWAFKRGAGINLKRVSADQSKDSRGKLLCNIND
CWH          529   YP_009206142.1      KVSKVIQEAKNQL-GKPYKWGGN-------------GPKSFDCSGLMVWAFKRGAGINLKRVSADQSKDSRGKLLCNIND
Acd24020     271   CAJ69287.1          GADSVISFAKTLL-GKPYVWGAE-------------GPNSFDCSGFTQYVMKKSVGVSIPRTAQAQYDAT---QHLPLSQ
CD03720      213   CAJ67194.1          LAQKVMNEALKYQGWKYVYGGSN-------------PNTSFDCSGLTQWCY-GKAGISLPRVSRDQSKYG---TYVNRGD
NpPCP         97   ZP_00105875.2       LAEVIAFTQKAMQQSNYYLWGGT-------------VGPNYDCSGLMQAAF-ASVGIWLPRDAYQQEGFT---QPITIAE
RipA         342   4Q4G                ASEYVIRRGMSQI-GVPYSWGGGNAAGPSKGIDSGAGTVGFDASGLVLYSF-AGVGIKLPHYSGSQYNLG---RKIPSSQ
P60_tth      127   BAD70089.1          PESPLLRAVLRYL-GVPYKYGAN-------------SPLALDCSAFVAQVY-AELGVALPRTTKEQYQ-----AFPPVEA
BcYkfC       188   3H41                AADDLINTGKMFL-GLPYIWAGT-------------SGFGFDCSGFTHTIY-KSHGITIPRDSGPQSRNG---VAVDKEH

                      Accession no.
Ecd09610     596   CAJ67802.1          VKAGDLVFFAYN-KGKGNVHHVGLYIGNDQYIHAPQTGD---VVKISSLSG---R--QKKKHDFARARRFF----
CWH          596   YP_009206142.1      VKAGDLVFFAYN-KGKGNVHHVGLYIGNDQYIHAPQTGD---VVKISSLSG---R--QKKKHDFARARRFF----
Acd24020     337   CAJ69287.1          LRSGDLVFFDTQGSNNGSVSHVGIYIGNGDMIHASSGSSK-DPIGYAD------LSSSYWQQHLIGAGRVKQ---
CD03720      276   CAJ67194.1          AKAGDLVFFHSTYNAGSYVTHVGIYVGNNQMYHAG-------KVTISNINS------SYYSSRYVNARRVL----
NpPCP        161   ZP_00105875.2       LVAGDLVFFGTSQK----ATHVGLYLADGYYIHSSGKDQGRDGIGIDILSEQGDAVSLSYYQQLRGAGRVFKS--
RipA         418   4Q4G                MRRGDVIFYGPNG-----SQHVTIYLGNGQMLEAPDVGLK---VRVAPVRT---AGMTPYVVRYIEY--------
P60_tth      188   BAD70089.1          PRPGDLVFFSFGG---KEVDHVGIYLGRGVFAHASSYGSR---VVIESLEA------PFYRKVYRGARRVMASPE
BcYkfC       251   3H41                LQKGDLIFFAHD-QGKGSVHHVAMYIGDGNMIHSPRAE-R--SVEIIPLNT------PGYIEEYAGARRYLP---
```

Figure S1. Multiple sequence alignment of the catalytic domains of Ecd09610.

(a) N-acetylglucosaminidase (PF01832: GH73 family) and (b) endopeptidase (PF00877: NlpC/P60 family) are shown. The multiple sequence alignment was created using Clustal Omega (https://www.ebi.ac.uk/Tools/msa/clustalo/). Accession no. indicates the NCBI GenBank accession number. Conserved and identical residues (more than half) are shaded in gray and black, respectively.

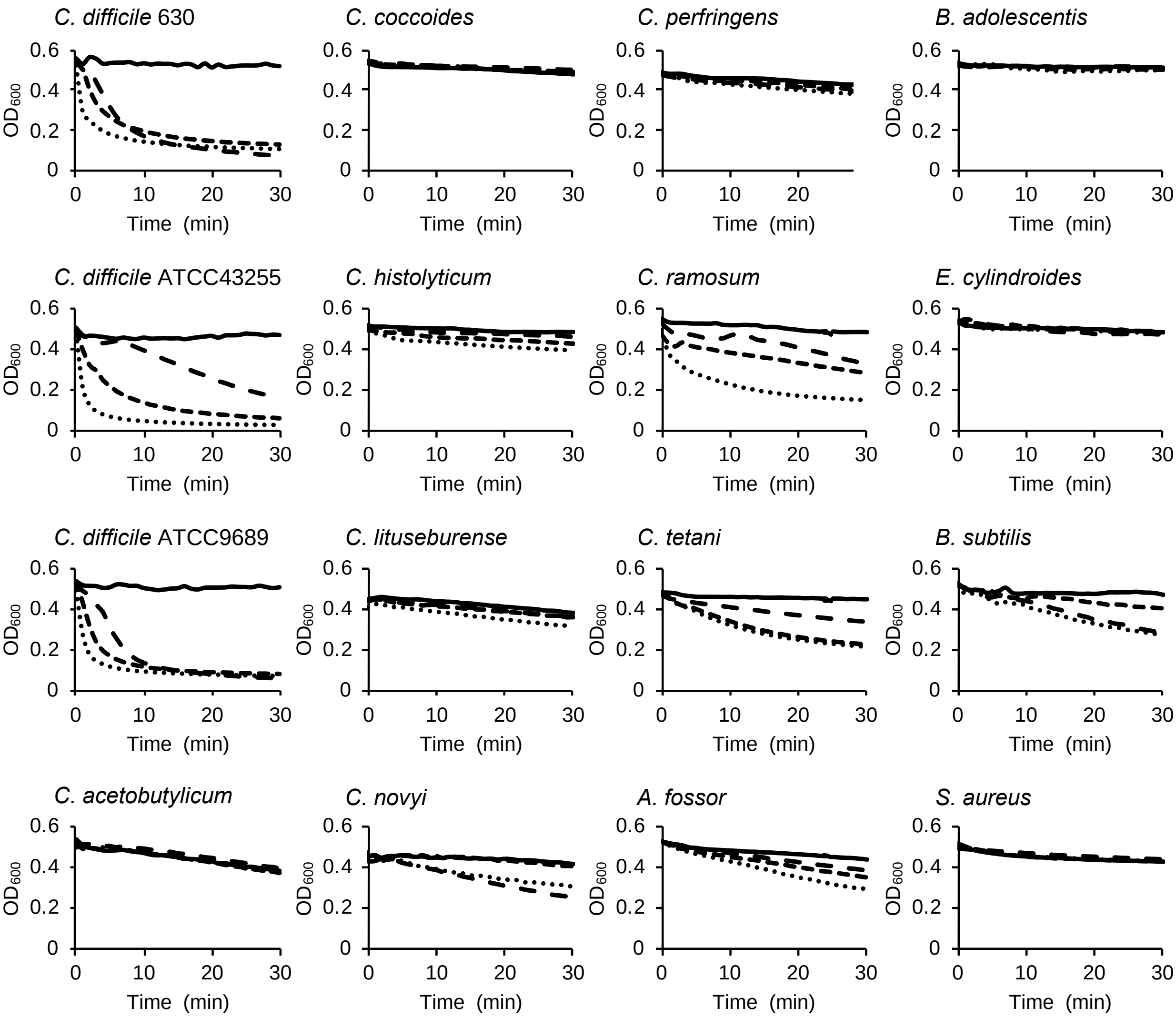

Figure S2. Species specificity of Ecd09610CD1, Ecd09610CD3, and Ecd09610CD53 lytic activities.

Experiments were repeated three times with similar results. Ecd09610CD1 (0.14 μM, dotted line), Ecd09610CD3 (0.2 μM, dashed line), or Ecd09610CD53 (0.3 μM, long dashed line) or buffer (solid line) was added to the cells, and OD_{600} was measured at 1-min intervals for 30 min.

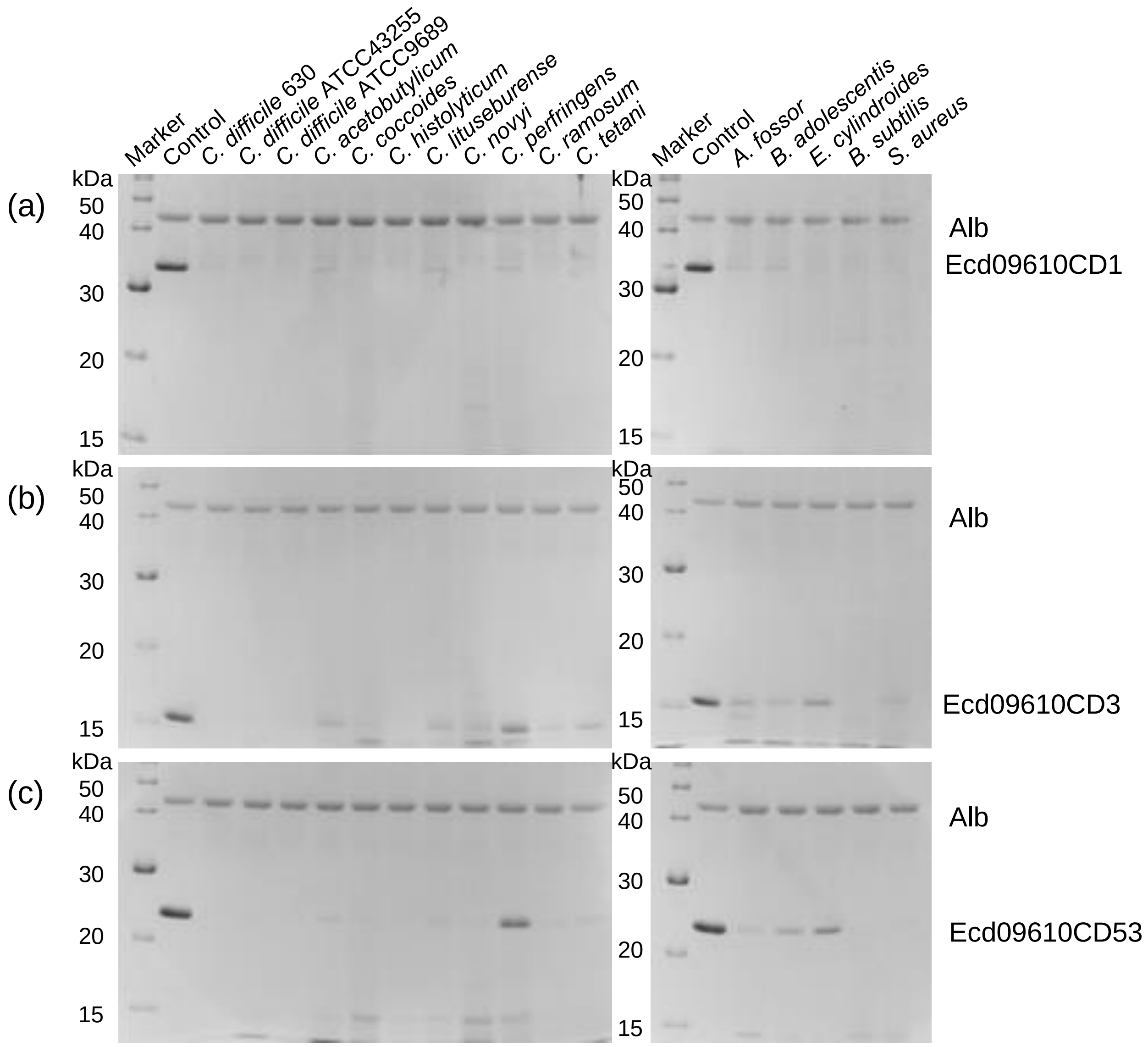

Figure S3. Binding assay for Ecd09610CD1, Ecd09610CD3, and Ecd09610CD53 with various bacteria. Experiments were repeated three times with similar results. One microgram of purified Ecd09610CD1 (a), Ecd09610CD3 (b), Ecd09610CD53 (c), or ovalbumin (Alb) as a nonbinding internal standard were incubated on ice with or without heat-inactivated cells as indicated at the top of the gel. After centrifugation, the supernatants were analyzed by 13.5% SDS-PAGE.

a

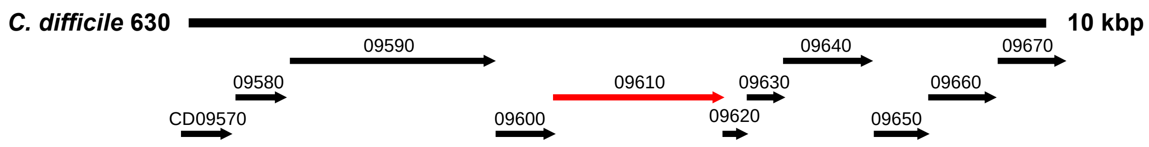

b

Gene Tag	aa	Description
CD09570	186	Putative phage lipoprotein
CD09580	189	Putative phage protein
CD09590	783	Putative phage tail tape measure protein
CD09600	229	Putative phage cell wall hydrolase
CD09610	656	Putative phage cell wall hydrolase
CD09620	86	Putative phage protein
CD09630	139	Putative phage protein
CD09640	349	Putative phage baseplate assembly protein
CD09650	205	Putative phage protein
CD09660	267	Putative phage tail fiber protein
CD09670	568	Putative phage protein

Figure S4. Genes around ecd09610 (CD09610) in the *C. difficile* 630 genome.

(a) *C. difficile* 630 has a group of phage-rerated genes of about 60 kbp that constitute a phage remnant region.

(b) Genes in the genome region from CD09570 to CD09670 in *C. difficile* 630 are shown.

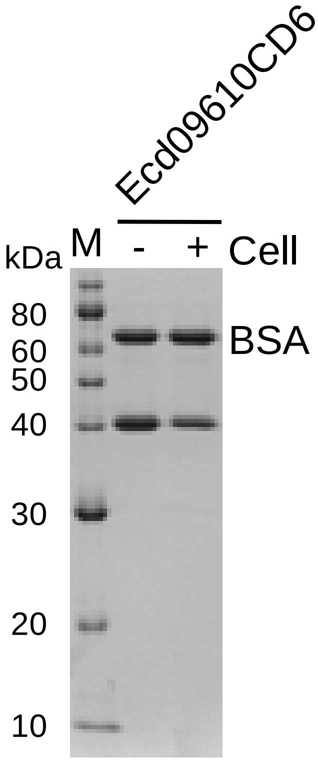

Figure S5. Binding ability of the purified N-terminal uncharacterized region of Ecd09610 to *C. difficile* 630 cells.

One microgram of purified Ecd09610CD6, which has only the N-terminal uncharacterized region of Ecd09610, and nonbinding internal standard (bovine serum albumin: BSA) were incubated with or without heat-inactivated cells on ice. After centrifuging the samples, the supernatants were analyzed by 13.5% SDS-PAGE.

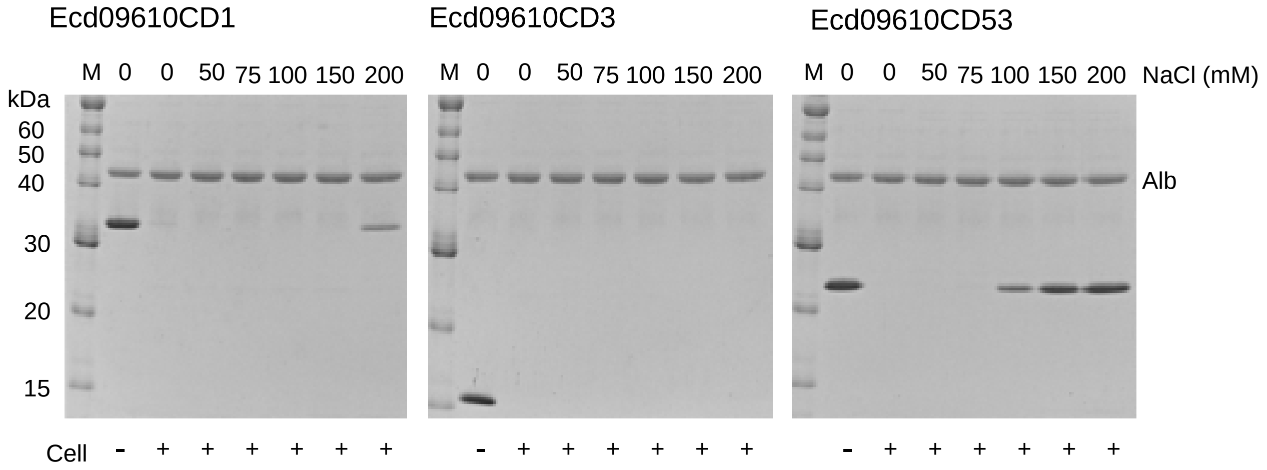

Figure S6. Effect of NaCl on the binding ability of purified proteins to *C. difficile* 630.

The binding abilities of Ecd09610CD1, Ecd09610CD3, and Ecd09610CD53 to *C. difficile* 630 are shown. One microgram of purified Ecd09610CD1, Ecd09610CD3, Ecd09610CD53, and nonbinding internal standard (ovalbumin: Alb) were incubated with or without heat-inactivated cells on ice. After centrifuging the samples, the supernatants were analyzed by 13.5% SDS-PAGE.

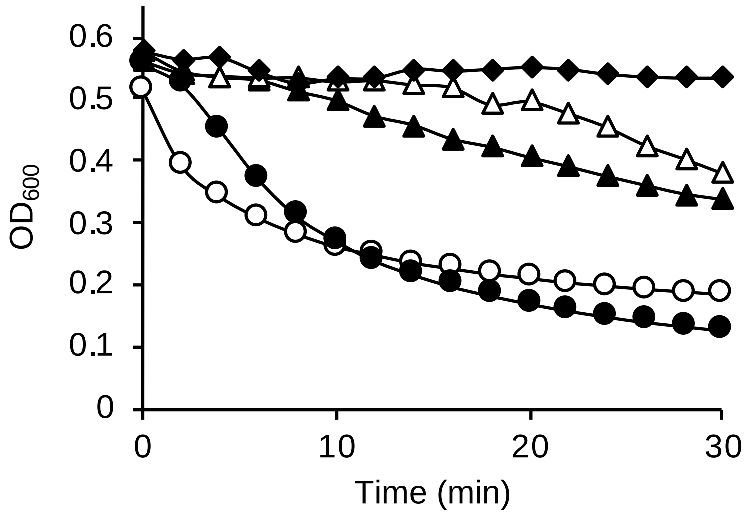

Figure S7. Effect of the two catalytic domains.

The lytic activities of proteins (0.08 μM) were determined by the turbidity reduction assay against *C. difficile* 630 cells. Both Ecd09610CD3 and Ecd09610CD53 together (filled circles), Ecd09610CD1 (open circles), Ecd09610CD3 (filled triangles), Ecd09610CD53 (open triangles), and control (filled diamonds) are shown.

Table S1 Primers used in this study.

Primer name	Primer sequence (5'-3')
CD09610-N	CGCcatatgGTTGATGAATTAGTGTTAG
CD09610-m1	CCCCAAGCATACGGCTTACCAAG
CD09610-m2	CTTGGTAAGCCGTATGCTTGGGG
CD09610-C	CGCggatccTTAAAAGAATCTTCTAGCTCTTG
CD09610-CD1	CCCcatatgACCACAAGTAAAGAAGACAATG
CD09610-CD3	CCCcatatgAAAGTAAGTAAAGTTATTCAAGAAGC
CD09610-CD5 R	CCCggatccTTAATACGGCTTACCAAGTTG
pCold F	ACGCCATATCGCCGAAAGG
pCold R	TGGCAGGGATCTTAGATTCTG
